# Supplementary material for: Somatic cells compartmentalise their metabolism to sustain germ cell survival
Source: bioRxiv. 2025 Jul 24:2025.07.22.666113. Preprint. [Version 3] doi: 10.1101/2025.07.22.666113 (PMC12330681; doi:10.1101/2025.07.22.666113)

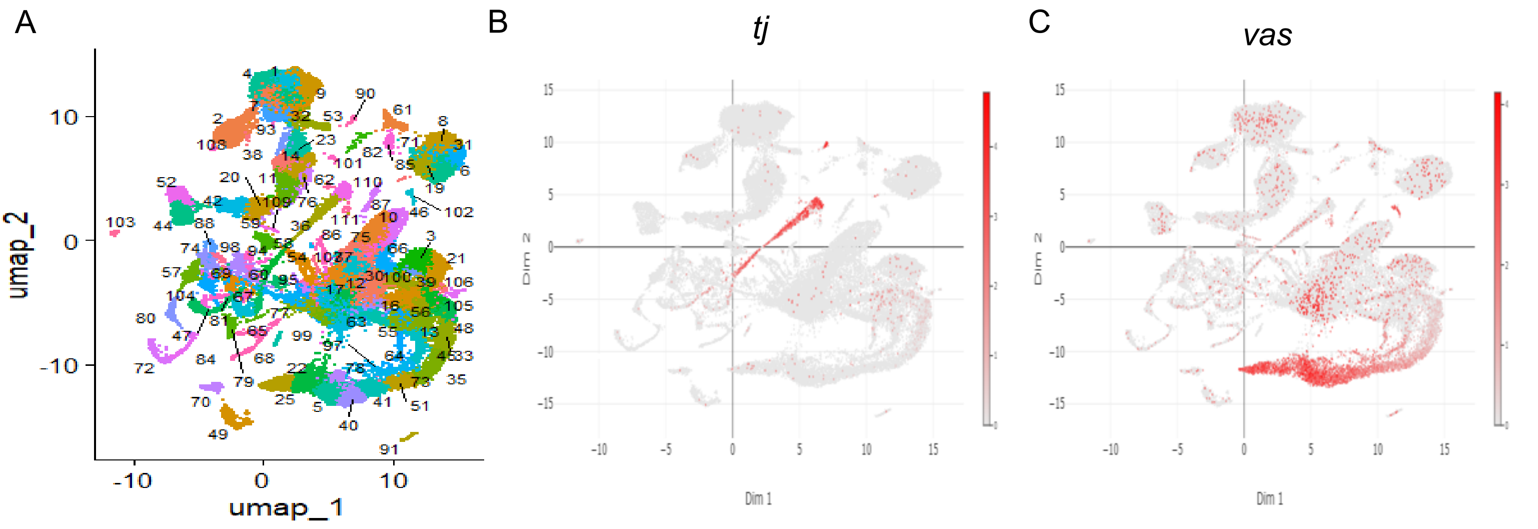

D

Comparison of somatic clusters 62, 36, 58 vs germ clusters 25 and 22										
GO term (FDR value)	Genes									
Biosynthesis of amino acids (FDR=0,003)	*Eno*	*Gapdh2*	*Pfk*	*Pgam1*	*Tpi*	*aay*	*Alat*	*Arg*	*Bcat*	*CG3483*
	CG32026	*Glts*	*Gs1*	*Gs2*	*Idh*	*Idh3g*	*Irp-1B*	*P5CS*	*Pcb*	*Phgdh*
	Psat	*Pykl2*	*Pykl5*	*Sams*	*Taldo*	*Tkt*	*Tktl*			
Carbon metabolism (FDR=0,003)	*Eno*	*Gapdh2*	*Hex-A*	*Pfk*	*Pgam1*	*Tpi*	*aay*	*Acat1*	*Acat2*	*AcCoAS*
	Alat	*CG3483*	*CG5577*	*CG17544*	*CG17896*	*CG32026*	*CG32487*	*CG32488*	*Fbp*	*G6pd*
	G6pdl	*Gdh*	*Hibadh*	*Idh*	*Idh3g*	*Irp-1B*	*Mdh1*	*Mdh2*	*Ogdh1*	*Pcb*
	Pgd	*Phgdh*	*Psat*	*Pykl2*	*Pykl5*	*SdhA*	*SdhAL*	*Taldo*	*Tkt*	*Tktl*

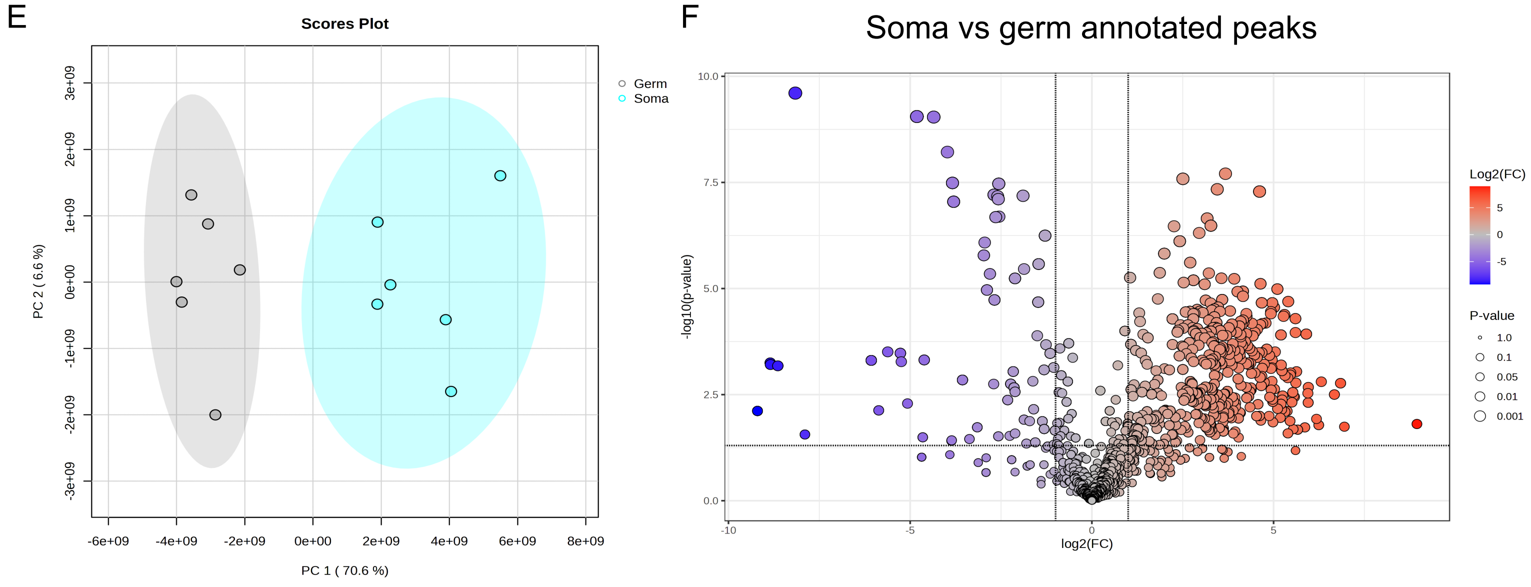

Supplement: Supplement 2 — Figure S1. Somatic and germ cells have different metabolic profiles (A) UMAP representation showing the clustering and annotation generated by Raz et al of the Fly Cell Atlas single-nucleus RNA sequencing of the testis. (B) Gene expression plot showing tj expression on the testis UMAP identifying clusters 62, 36 and 58 as corresponding to CySCs and early cyst cells. (C) Gene expression plot showing vasa (vas) expression on the testis UMAP representation, identifying clusters 25, 22 and 5 as corresponding to early stages of germ cell development. (D) Table listing the two most significantly enriched GO terms in genes enriched in early cyst cells (clusters 62, 36 and 58) compared to early germline clusters (25 and 22). The genes contained within the GO categories are shown, and those encoding glycolytic enzymes are highlighted in yellow. (E) Principal component analysis score plot from the mass spectrometry data from the independent replicates of sorted cyst (blue dots) and germ cells (grey dots). (F) Volcano plot showing the relative enrichment of all annotated mass spectrometry peaks in cyst cells compared to germ cells. [file media-2.pdf]
