# Supplementary figures and images for: Somatic cells compartmentalise their metabolism to sustain germ cell survival
Source: bioRxiv. 2025 Jul 24:2025.07.22.666113. Preprint. [Version 3] doi: 10.1101/2025.07.22.666113 (PMC12330681; doi:10.1101/2025.07.22.666113)

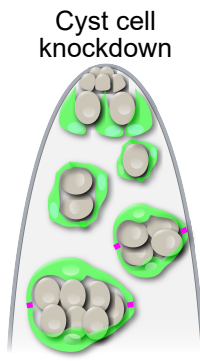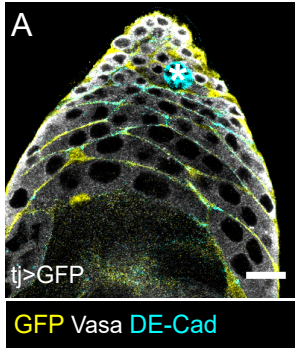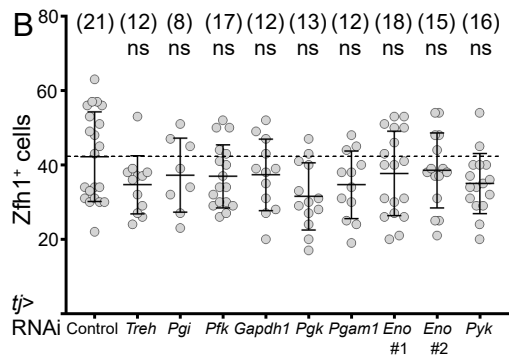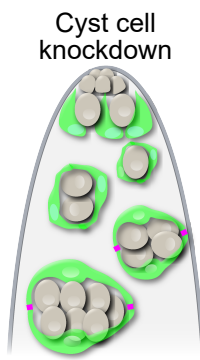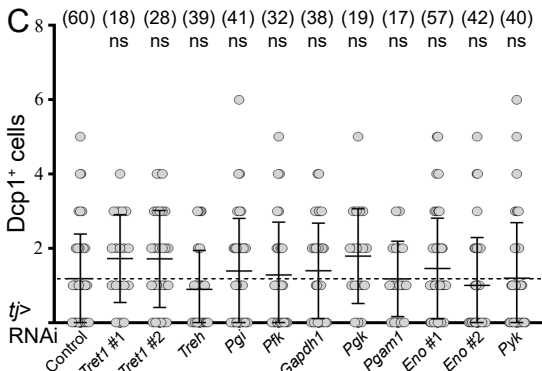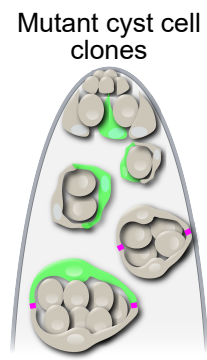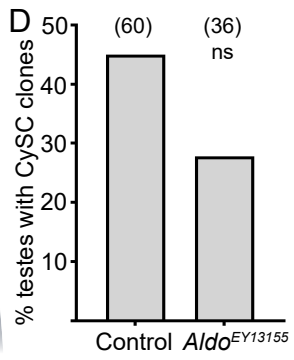

Supplement: Supplement 3 — Figure S2. Knockdown of genes encoding glycolytic enzymes does not affect CySC numbers or cyst cell survival. (A) Confocal image of a testis expressing GFP (yellow) in somatic cells driven by tj-Gal4. Vasa (white) labels germ cells, DE-Cad (cyan) labels cell outlines. Scale bar: 20 μm. The diagram on the left represents a testis apical tip and highlights tj-Gal4 expression in green. (B) Graph showing the number of Zfh1-positive, Eya-negative cells in control testes or testes in which the indicated genes encoding glycolytic enzymes were knocked down in cyst cells. Significance was assessed using Kruskal Wallis and Dunn’s multiple comparisons tests. (C) Graph showing the number of Dcp-1-positive cells in control testes or testes in which the indicated genes encoding glycolytic enzymes were knocked down in cyst cells. Significance was assessed using Kruskal Wallis and Dunn’s multiple comparisons tests. (D) Graph showing the recovery rate of control and AldoEY13155 somatic clones. No significant difference was detected using Fisher’s exact test. All graphs: N values are shown in brackets and refer to the number of testes analysed. [file media-3.pdf]

Early germ cell  
knockdown

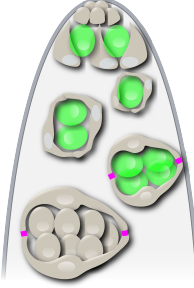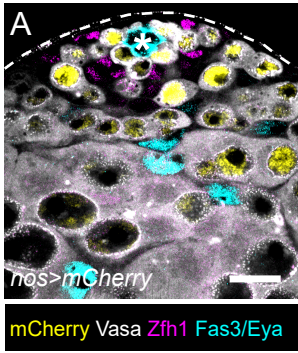

Late germ cell  
knockdown

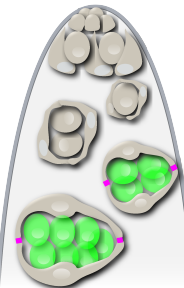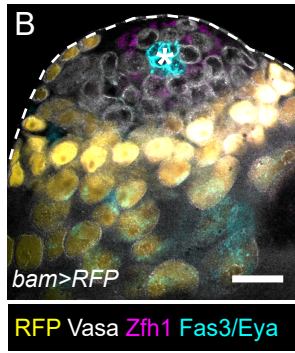

Early germ cell  
knockdown

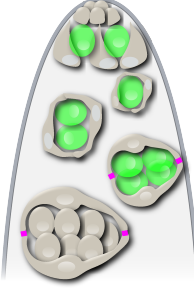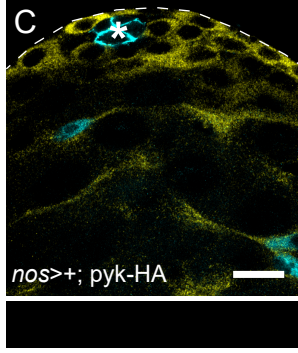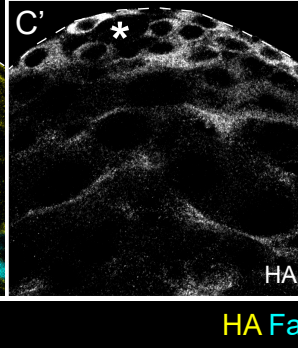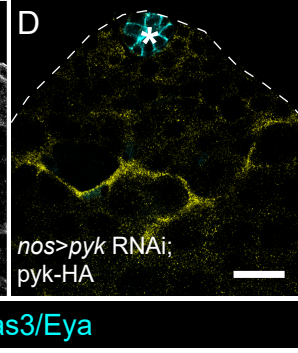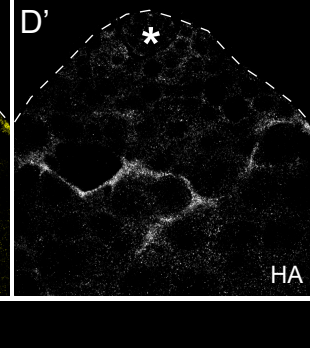

Supplement: Supplement 4 — Figure S3. Driver expression and RNAi knockdown in the germline. (A,B) Confocal images of testes expressing mCherry (yellow) in early germ cells cells driven by nos-Gal4 (A) or RFP (yellow) in differentiating germ cells driven by bam-Gal4 (B). Vasa (white) labels germ cells, Zfh1 (magenta) labels CySCs, Fas3 and Eya (cyan) label the hub and cyst cells, respectively. Scale bar: 20 μm. The diagrams on the left represent a testis apical tip and highlight the domains of nos-Gal4 and bam-Gal4 expression in green. (C,D) Confocal images of testes from Pyk-HA control flies (C) or flies in which Pyk was knocked down in early germ cells with nos-Gal4, labelled with antibodies against HA (yellow) and Fas3 and Eya (cyan) to label the hub and cyst cells respectively. Scale bars: 20 μm. [file media-4.pdf]

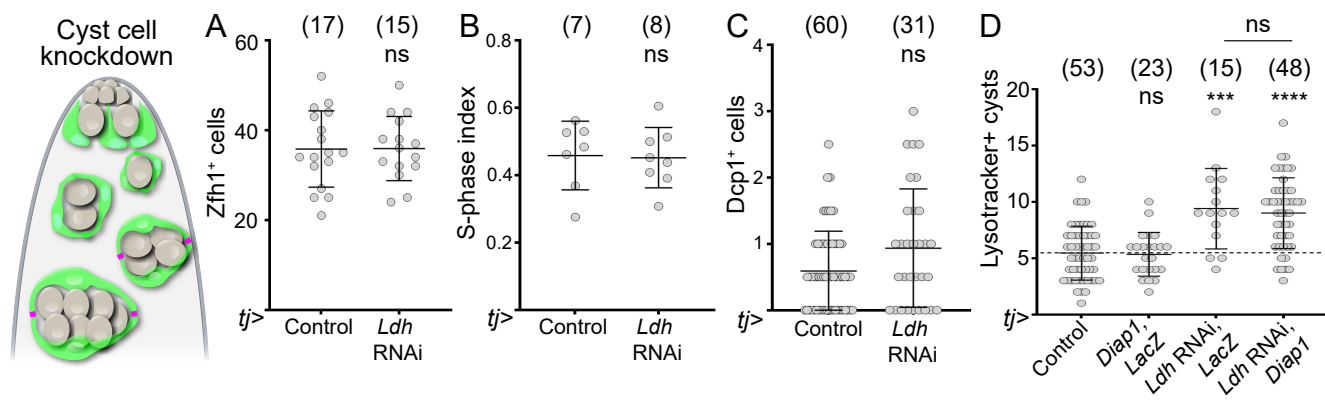

Supplement: Supplement 5 — Figure S4. Ldh is not required autonomously in CySCs or cyst cells. (A) Graph showing the number of Zfh1-positive, Eya-negative cells in control testes or testes in which Ldh was knocked down in cyst cells. Significance was assessed using a Mann-Whitney test. (B) S-phase index of Zfh1-positive CySCs in control testes and testes in which Ldh was knocked down in cyst cells. Significance was assessed using a student’s t test. (C) Graph showing the number of Dcp-1-positive cells in control testes and testes in which Ldh was knocked down in cyst cells. Significance was assessed using a Mann-Whitney test. (D) Graph showing the number of Lysotracker-positive germ cell cysts in control testes and testes in which Ldh was knocked down in cyst cells, with or without overexpression of the apoptosis inhibitor Diap. *** denotes P < 0.001, **** denotes P < 0.0001, determined by Kruskal Wallis and Dunn’s multiple comparisons tests. All graphs: N values are shown in brackets and refer to the number of testes analysed. [file media-5.pdf]

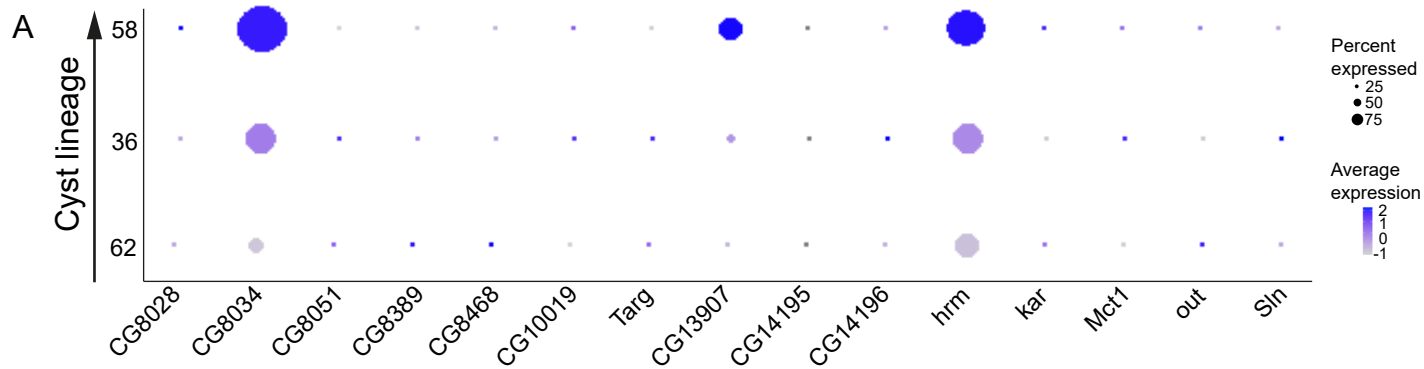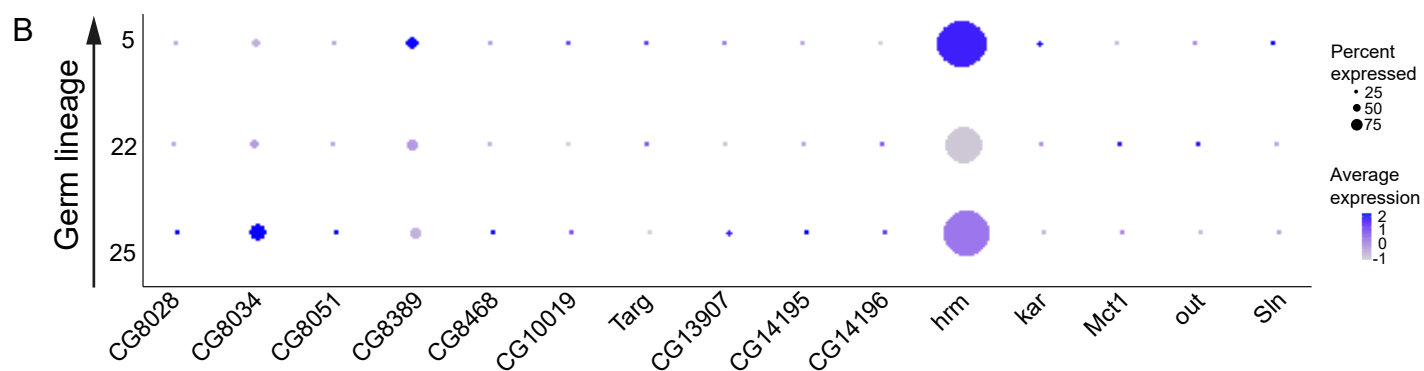

Supplement: Supplement 6 — Figure S5. Expression of monocarboxylate transporters in somatic and germ lineages. (A) Dot plot showing average expression from the Fly Cell Atlas dataset of the indicated genes encoding MCTs in the cell clusters corresponding to CySCs (62) and early cyst cells (36 and 58). (B) Dot plot showing average expression from the Fly Cell Atlas dataset of the indicated genes encoding MCTs in the cell clusters corresponding to early (25), and late spermatogonia (22) and cells at the spermatogonia-spermatocyte transition (5). The size of the dots indicates the percent of expressing cells in each cluster, and the colour represents the expression level. Note that chaski was not identified in this dataset. [file media-6.pdf]

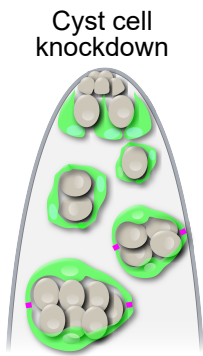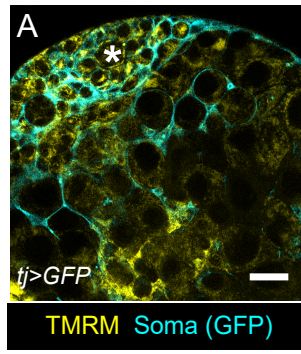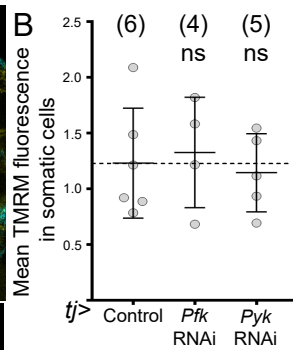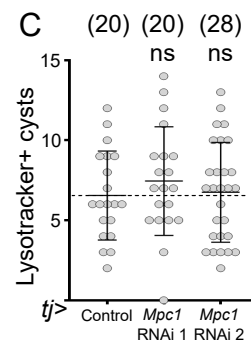

Supplement: Supplement 7 — Figure S6. Mitochondrial activity in cyst cells does not depend on autonomous pyruvate consumption. (A) Airyscan image of a testis in which GFP (cyan) was expressed with tj-Gal4 to identify cyst cells and labelled with the mitochondrial membrane potential-sensitive dye TMRM (yellow). Scale bar: 20 μm. (B) Graph showing TMRM mean intensity in cyst cells in control testes and testes in which Pfk or Pyk were knocked down. Significance was determined by Kruskal Wallis and Dunn’s multiple comparisons tests. (C) Graph showing the number of Lysotracker-positive cysts in control testes and testes in which Mpc1 was knocked down in cyst cells. Significance was determined by Kruskal Wallis and Dunn’s multiple comparisons tests. All graphs: N values are shown in brackets and refer to the number of testes analysed. [file media-7.pdf]
